# Simultaneous identification of 36 mutations in *KRAS* codons 61and 146, *BRAF*, *NRAS*, and *PIK3CA* in a single reaction by multiplex assay kit

**DOI:** 10.1186/1471-2407-13-405

**Published:** 2013-09-03

**Authors:** Hideaki Bando, Takayuki Yoshino, Eiji Shinozaki, Tomohiro Nishina, Kentaro Yamazaki, Kensei Yamaguchi, Satoshi Yuki, Shinya Kajiura, Satoshi Fujii, Takeharu Yamanaka, Katsuya Tsuchihara, Atsushi Ohtsu

**Affiliations:** 1 Department of Gastroenterology and Gastrointestinal Oncology, National Cancer Center Hospital East, 6-5-1 Kashiwanoha, Kashiwa, Chiba 277-8577, Japan; 2 Cancer Institute Hospital of Japanese Foundation for Cancer Research, Tokyo, Japan; 3 National Hospital Organization Shikoku Cancer Center, Ehime, Japan; 4 Division of Gastrointestinal Oncology, Shizuoka Cancer Center, Shizuoka, Japan; 5 Division of Gastroenterology, Saitama Cancer Center, Saitama, Japan; 6 Department of Gastroenterology, Hokkaido University Graduate School of Medicine, Hokkaido, Japan; 7 The Third Department of Internal Medicine, University of Toyama, Toyama, Japan; 8 Pathology Division, Research Center for Innovative Oncology, National Cancer Center Hospital East, Chiba, Japan; 9 Exploratory Oncology Research & Clinical Trial Center, National Cancer Center, Chiba, Japan

**Keywords:** Luminex assay, *KRAS*, *BRAF*, *NRAS*, *PIK3CA*, Epidermal growth factor

## Abstract

**Background:**

Retrospective analyses in the West suggest that mutations in *KRAS* codons 61 and 146, *BRAF*, *NRAS*, and *PIK3CA* are negative predictive factors for cetuximab treatment in colorectal cancer patients. We developed a novel multiplex kit detecting 36 mutations in *KRAS* codons 61 and 146, *BRAF*, *NRAS*, and *PIK3CA* using Luminex (xMAP) assay in a single reaction.

**Methods:**

Tumor samples and clinical data from Asian colorectal cancer patients treated with cetuximab were collected. We investigated *KRAS, BRAF*, *NRAS*, and *PIK3CA* mutations using both the multiplex kit and direct sequencing methods, and evaluated the concordance between the 2 methods. Objective response, progression-free survival (PFS), and overall survival (OS) were also evaluated according to mutational status.

**Results:**

In total, 82 of 83 samples (78 surgically resected specimens and 5 biopsy specimens) were analyzed using both methods. All multiplex assays were performed using 50 ng of template DNA. The concordance rate between the methods was 100%. Overall, 49 (59.8%) patients had all wild-type tumors, 21 (25.6%) had tumors harboring *KRAS* codon 12 or 13 mutations, and 12 (14.6%) had tumors harboring *KRAS* codon 61, *KRAS* codon 146, *BRAF*, *NRAS*, or *PIK3CA* mutations. The response rates in these patient groups were 38.8%, 4.8%, and 0%, respectively. Median PFS in these groups was 6.1 months (95% confidence interval (CI): 3.1–9.2), 2.7 months (1.2–4.2), and 1.6 months (1.5–1.7); median OS was 13.8 months (9.2–18.4), 8.2 months (5.7–10.7), and 6.3 months (1.3–11.3), respectively. Statistically significant differences in both PFS and OS were found between patients with all wild-type tumors and those with *KRAS* codon 61, *KRAS* codon 146, *BRAF*, *NRAS*, or *PIK3CA* mutations (PFS: 95% CI, 0.11–0.44; *P* < 0.0001; OS: 95% CI, 0.15–0.61; *P* < 0.0001).

**Conclusions:**

Our newly developed multiplex kit is practical and feasible for investigation of a range of sample types. Moreover, mutations in *KRAS* codon 61, *KRAS* codon 146, *BRAF*, *NRAS*, or *PIK3CA* detected in Asian patients were not predictive of clinical benefits from cetuximab treatment, similar to the result obtained in European studies.

## Background

The clinical significance of *KRAS* codon 12 and 13 mutation tests in the selection of patients with colorectal cancer who might benefit from anti-epidermal growth factor receptor (EGFR) antibodies is well established, and regulatory authorities in Europe, the United States, and Japan have recommended compulsory *KRAS* mutation testing before treatment [1-6]. Although conventional *KRAS* tests are useful to decrease treatment to nonbeneficiary populations, the efficacy of determining beneficiary populations requires improvement. The response rate to anti-EGFR antibody monotherapy among pretreated patients with tumors harboring *KRAS* codons 12 and 13 wild-type is 13%–17% [1,2], and that of combination anti-EGFR antibody and cytotoxic agent therapy is 11%–35% [5,7]. One explanation for such relatively low efficacy is that molecular alterations other than *KRAS* codon 12 and 13 mutations might confer resistance to anti-EGFR antibody therapies. Recent retrospective studies have revealed that mutations in *KRAS* codons 61 and 146, *BRAF*, *NRAS*, and *PIK3CA* are also related to resistance to anti-EGFR antibodies [8-13].

Several issues should also be considered to establish the clinical utility of expanded genome biomarker tests for anti-EGFR antibodies. First, information about the relation between mutation status and efficacy of treatment, especially among Asian populations, is still limited. Second, efficacious quality-controlled *in vitro* diagnostic kits and systems suitable for multiple genome biomarker detection are needed.

In Japan, a *KRAS* mutation assay kit based on the ARMS–scorpion method that detects seven frequently observed mutations in *KRAS* codons 12 and 13 (TheraScreen® K-RAS Mutation Kit; QIAGEN) was first approved for *in vitro* diagnostic use, and a kit using Luminex (xMAP) assay (MEBGEN KRAS Mutation Detection Kit, MBL) followed [14,15]. We recently developed another Luminex-based research-use kit, GENOSEARCH Mu-PACK, which simultaneously detects 36 mutations in *KRAS* codons 61 and 146, *BRAF*, *NRAS*, and *PIK3CA*. In addition to the hitherto approved *KRAS* codon 12 and 13 mutation kit, the multiplex kit identifies mutations by a single tube reaction using 50 ng of template DNA from formalin-fixed paraffin-embedded (FFPE) specimens.

In this study, we examined the feasibility and robustness of this multiplex kit using routine clinical samples collected from multiple hospitals. Meanwhile, we collected precise clinical data for these cases and retrospectively analyzed the relation of the mutation profiles of expanded markers to clinical outcomes following cetuximab therapy.

## Methods

### Patients

We screened and selected clinical and pathological data from consecutive patients who were administered either cetuximab monotherapy or cetuximab plus irinotecan between July 2008 and April 2010.

Patients who met all of the following inclusion criteria were retrospectively included in the analyses: (1) age ≥20 years; (2) histologically confirmed adenocarcinoma of the colon or rectum; (3) presence of unresectable metastatic disease; (4) baseline computed tomography (CT) performed within 28 days of initial cetuximab administration; (5) initial CT evaluation performed within 3 months of initial cetuximab administration; (6) previously documented as refractory or intolerant to fluoropyrimidines, oxaliplatin, and irinotecan; (7) Eastern Cooperative Oncology Group performance status score ≤2; and (8) adequate hematological, hepatic, and renal functions.

In the monotherapy regimen, cetuximab was administered at an initial dose of 400 mg/m^2^ followed by weekly infusions of 250 mg/m^2^. In the cetuximab plus irinotecan regimen, cetuximab was administered at the same dose as for monotherapy and followed by biweekly infusions of 150 mg/m^2^ irinotecan, as per the manufacturer’s instructions for irinotecan in Japan.

The study was conducted with the approval of the National Cancer Center Institutional Review Board, Cancer Institute Hospital of Japanese Foundation for Cancer Research Review Board, National Hospital Organization Shikoku Cancer Center Review Board, Shizuoka Cancer Center Review Board, Saitama Cancer Center Review Board, Hokkaido University Review Board, and the Ethics Committee of the University of Toyama. Written informed consent was obtained from as much patients who were alive as possible. For the deceased patients and their relatives, we also disclosed the study design at the website of National Cancer Center and gave them chances to express their wills in accordance with Epidemiological Study Guideline of Ministry of Health, Labour and Welfare in Japan.

### Tissue samples and DNA extraction

Genomic DNA was obtained from primary and metastatic colorectal cancer tissues of all patients treated with cetuximab. Tissue samples harvested by biopsy or surgical resection at the participating hospitals were collected and sent to the research institution (MBL, Japan). A 2-μm hematoxylin-eosin (HE) slide and a 10-μm unstained slide were obtained from the FFPE tissue blocks; the latter was subsequently sliced into 3–10 sections. Pathological diagnoses were confirmed by a pathologist (Satoshi Fujii), with reference to the 4^th^ edition of the WHO classification. The tumor area, determined by examining HE slides, was macroscopically dissected. Genomic DNA was isolated as described previously [16].

### Luminex (xMAP) tests

A total of 36 mutations of *KRAS* codon 61 (Q61K, Q61E, Q61L, Q61P, Q61R, Q61H), *KRAS* codon 146 (A146T, A146S, A146P, A146E, A146V, A146G), *BRAF* codon 600 (V600E), *NRAS* codon 12 (G12S, G12C, G12R, G12D, G12V, G12A), codon 13 (G13S, G13C, G13R, G13D, G13V, G13A), codon 61 (Q61K, Q61E, Q61L, Q61P, Q61R, Q61H), *PIK3CA* exon 9 codon 542 (E542K), codon 545 (E545K), codon 546 (E546K), and exon 20 codon 1047 (H1047R, H1047L) were analyzed using Luminex (xMAP) technology (GENOSEARCH Mu-PACK, MBL, Japan).

First, 50 ng of template DNA collected from FFPE tissue samples was amplified by polymerase chain reaction (PCR) using a biotin-labeled primer. Thereafter, the PCR products and fluorescent Luminex beads (oligonucleotide probes complementary to wild and mutant genes were bound to the beads) were hybridized and labeled with streptavidin–phycoerythrin. Subsequently, the products were processed by Luminex assay and the collected data analyzed using UniMAG software (MBL, Japan). The procedure time was approximately 4.5 h.

We also used the Luminex assay kit (MEBGEN KRAS Mutation Detection Kit, MBL, Japan) currently approved for clinical use by the Ministry of Health, Labour and Welfare of Japan [16] to detect *KRAS* codon 12 and 13 mutations.

### Direct sequencing methods

In addition, to confirm the mutations detected by the Luminex assays, the same mutations of *KRAS* codons 61 and 146, *BRAF*, *NRAS*, and *PIK3CA* were analyzed by direct sequencing. A total of 700 ng of template DNA was used for these PCR reactions and the PCR products were directly sequenced with the same primers used for PCR. A BigDye Terminator v3.1 Cycle Sequencing Kit and an ABI PRISM 3730xl DNA Analyzer (Life Technologies) were used. Analyses of DNA sequences were performed using Sequencher (GeneCodes).

### Statistical analysis

Response rates (RRs) and disease control rates (DCRs) (including complete or partial response and stable disease) were evaluated as per the Response Evaluation Criteria in Solid Tumors (RECIST) (version 1.0). Progression-free survival (PFS) was defined as the time from initial administration of a cetuximab-containing regimen to either the first objective evidence of disease progression or death from any cause. Overall survival (OS) was defined as the time from initial administration of a cetuximab-containing regimen to death from any cause. RRs, DCRs, PFS, and OS of all patients were re-evaluated by the principal investigators at each institution. The relative dose intensity was defined as the ratio of the actual dose administered to the planned dose.

Fisher’s exact test and the Kruskal–Wallis test were used to compare patient characteristics, relative dose intensity, and treatment response. PFS and OS data were plotted as Kaplan–Meier curves, and differences among the groups according to *KRAS*, *BRAF*, *NRAS*, and *PIK3CA* gene status were compared using the log-rank test and hazard ratio calculated from a Cox regression model with a single covariate. All analyses were performed by a biostatistician (Takeharu Yamanaka), using IBM SPSS® Statistics 21 package software (SPSS Inc., Tokyo, Japan).

## Results

### Concordance between Luminex and direct sequencing

From September 2008 to April 2010, 376 patients were treated with a cetuximab-containing regimen at seven institutions. Of these, 83 patients met the inclusion criteria and specimens were collected from them for analysis (232 patients did not meet the inclusion criteria and 61 specimens could not be collected). We collected 78 surgically resected specimens and 5 biopsy specimens, from which the median amount of template DNA collected was 25,114 ng (range: 2740–84,738) and 1691 ng (range:1469–2668), respectively (Table 1).

**Table 1:** Template DNA harvested from FFPE specimens

	**Surgically resected**	**Biopsy**	**Total**
Number of specimens	78	5	83
Total amount of template DNA (ng) [median (range)]	25,114 (2,740–84,738)	1,691 (1,469–2,668)	22,591 (1,469–84,738)
Amount of template DNA per slice (ng) [median (range)]	8,371 (914–28,246)	370 (154–889)	7,530 (154–28,246)

One patient’s gene status could not be detected by either Luminex or direct sequencing because DNA harvested from the resected metastatic liver specimens could not be amplified by PCR. In the remaining 82 patients, the concordance rate for mutations between the 2 methods was 100% (Table 2).

**Table 2:** Concordance between Luminex and direct sequencing

**Gene**	**Direct sequencing (DS)**	**Luminex**	**Concordance rate**	**Mutation rate**
***KRAS *codon 61**	**3**	**3**	**100%**	**3.6%**
Q61K	0	0	100%	0%
Q61E	0	0	100%	0%
Q61L	0	0	100%	0%
Q61P	0	0	100%	0%
Q61R	0	0	100%	0%
Q61H	3	3	100%	3.6%
***KRAS *codon 146**	**2**	**2**	**100%**	**2.4%**
A146T	2	2	100%	2.4%
A146S	0	0	100%	0%
A146P	0	0	100%	0%
A146E	0	0	100%	0%
A146V	0	0	100%	0%
A146G	0	0	100%	0%
***BRAF *codon 600**	**4**	**4**	**100%**	**4.9%**
V600E	4	4	100%	4.9%
***NRAS *codon 12**	**2**	**2**	**100%**	**2.4%**
G12S	0	0	100%	0%
G12C	0	0	100%	0%
G12R	0	0	100%	0%
G12D	2	2	100%	2.4%
G12V	0	0	100%	0%
G12A	0	0	100%	0%
***NRAS *codon 13**	**0**	**0**	**100%**	**0%**
G13S	0	0	100%	0%
G13C	0	0	100%	0%
G13R	0	0	100%	0%
G13D	0	0	100%	0%
G13V	0	0	100%	0%
G13A	0	0	100%	0%
***NRAS *codon 61**	**0**	**0**	**100%**	**0%**
Q61K	0	0	100%	0%
Q61E	0	0	100%	0%
Q61L	0	0	100%	0%
Q61P	0	0	100%	0%
Q61R	0	0	100%	0%
Q61H	0	0	100%	0%
***PIK3CA *Exon 9**	**1**	**1**	**100%**	**1.2%**
E542K	1	1	100%	1.2%
E545K	0	0	100%	0%
E546K	0	0	100%	0%
***PIK3CA *Exon 20**	**3**	**3**	**100%**	**3.7%**
H1047R	1	1	100%	1.2%
H1047L	2	2	100%	2.4%

**Figure 1:**
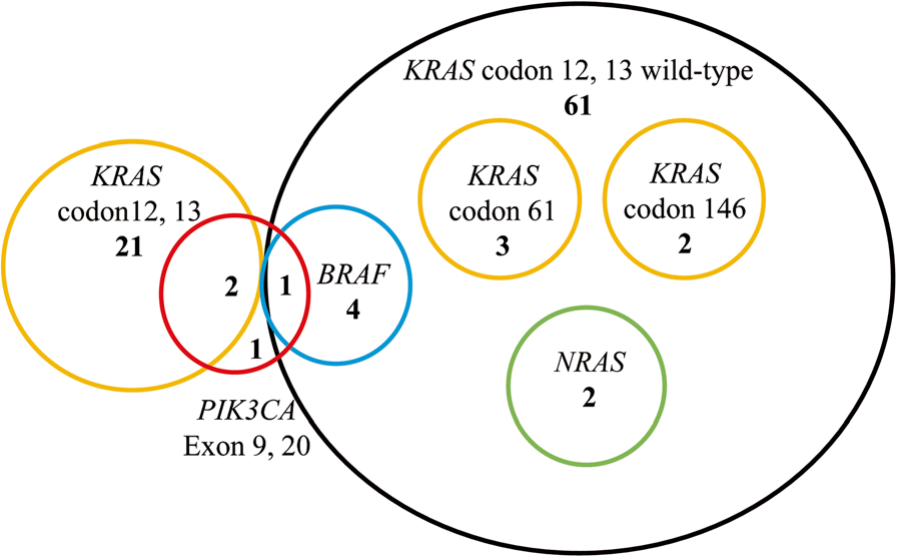
**Associations among *KRAS*, *BRAF*, *NRAS*, and *PIK3CA *mutations. *KRAS *codon 12 and 13, *KRAS *codon 61 and 146, *BRAF*, and *NRAS *mutations were mutually exclusive.** Only *PIK3CA* Exon 9 and 20 mutations overlapped *KRAS* codon 12 and 13 and *BRAF* mutations.

Among the 82 specimens, 3 *KRAS* codon 61 mutations (3.6%), 2 *KRAS* codon 146 mutations (2.4%), 4 *BRAF* mutations (4.9%), 2 *NRAS* mutations (2.4%), and 4 *PIK3CA* mutations (4.9%) (1 in exon 9 and 3 in exon 20) were detected using both the expanded kit and direct sequencing. Moreover, we identified 15 *KRAS* codon 12 mutations (18.3%) and 6 *KRAS* codon 13 mutations (7.3%); in total, 21 samples (25.6%) with *KRAS* codon 12 or 13 mutations were detected by using the *KRAS* Luminex assay kit. All mutations except for *PIK3CA* were mutually exclusive (Table 2, Figure 1).

### Patient characteristics

Clinical data were collected from 83 patients. We used data from 82 patients whose genomic DNA could be successfully examined using both the expanded kit and direct sequencing. Six of the 82 patients were treated with cetuximab monotherapy, while the remaining 76 were treated with a regimen of cetuximab plus irinotecan.

Of these 82 patients, 49 had tumors with no mutation (all wild type), 21 had tumors with mutation of either *KRAS* codon 12 or 13, and 12 had tumors with mutation of either *KRAS* codon 61, *KRAS* codon 146, *BRAF*, *NRAS*, or *PIK3CA*. No significant difference was observed in the characteristics of these three groups except for the ratio of refractoriness to intolerance of prior oxaliplatin (Table 3).

**Table 3:** Baseline patient characteristics

	**All wild-type**	***KRAS *codon 12, 13 mutations**	***KRAS *codon 61, codon 146, *BRAF*, *NRAS *or *PIK3CA *mutations (any other mutations)**	
	**(*N*** **= 49)**	**(*N*** **= 21)**	**(*N*** **= 12)**	
Treatment				
Cetuximab + irinotecan (%)	47 (96)	19 (90)	10 (83)	P = 0.212^†^
Cetuximab monotherapy (%)	2 (4)	2 (10)	2 (17)	
Age				
Median (range)	61 (29–78)	65 (51–80)	65 (43–76)	P = 0.605^‡^
Gender				
Male (%)	31 (63)	16 (76)	6 (50)	P = 0.312^†^
Female (%)	18 (37)	5 (24)	6 (50)	
ECOG PS				
0 (%)	34 (69)	13 (62)	5 (42)	P = 0.185^†^
1–2 (%)	15 (31)	8 (38)	7 (58)	
Primary lesion				
Colon (%)	28 (57)	15 (71)	9 (75)	P = 0.416^†^
Rectum (%)	21 (43)	6 (29)	3 (25)	
Site of Metastasis				
Liver				
Yes (%)	33 (67)	13 (62)	8 (67)	P = 0.945^†^
No (%)	16 (33)	8 (38)	3 (33)	
Lung				
Yes (%)	34 (69)	15 (71)	9 (75)	P = 1.000^†^
No (%)	15 (31)	6 (29)	3 (25)	
Lymph node				
Yes (%)	26 (53)	7 (33)	9 (75)	P = 0.068^†^
No (%)	23 (47)	14 (67)	3 (25)	
Peritoneum				
Yes (%)	11 (22)	3 (14)	2 (17)	P = 0.791^†^
No (%)	38 (78)	18 (86)	9 (83)	
No. of metastatic sites				
1 (%)	9 (18)	9 (42)	3 (25)	P = 0.106^†^
>2 (%)	40 (82)	12 (58)	9 (75)	
Prior chemotherapy				
Fluoropyrimidine				
Refractory (%)	49 (100)	21 (100)	12 (100)	
Intolerant (%)	0 (0)	0 (0)	0 (0)	
Oxaliplatin				
Refractory (%)	40 (82)	10 (48)	9 (75)	P = 0.017^†^
Intolerant (%)	9 (18)	11 (52)	3 (25)	
Irinotecan				P = 1.000^†^
Refractory (%)	48 (98)	21 (100)	12 (100)	
Intolerant (%)	1 (2)	0 (0)	0 (0)	P = 0.669^†^
Before bevacizumab therapy	25 (51)	9 (43)	7 (58)	
Yes (%)	24 (49)	12 (57)	5 (42)	P = 0.236^†^
No (%)	12	5	25	
Response rate for prior irinotecan-containing therapies (%)				
Pathological classification				
G1, G2 (%)	42 (86)	20 (95)	11 (92)	P = 0.481^†^
G3, G4 (%)	7 (14)	1 (5)	1 (8)	

*ECOG PS* Eastern Cooperative Oncology Group performance status.

^†^: Fisher’s exact test.

^‡^: Kruskal–Wallis test.

### Response to treatment

RRs of patients with all wild-type tumors (*N* = 49), *KRAS* codon 12 or 13 mutations (*N* = 21), and mutations of *KRAS* codon 61, *KRAS* codon 146, *BRAF*, *NRAS*, or *PIK3CA* (*N* = 12) were 38.8%, 4.8%, and 0%, respectively (Table 4). Partial response was observed in one patient with a *KRAS* codon G12C mutation. In addition, DCRs were 77.6%, 57.1%, and 33.3%, respectively, for these patient groups (Table 4). Differences for both RRs and DCRs between patients with all wild-type tumors and those with *KRAS* codon 61, *KRAS* codon 146, *BRAF*, *NRAS*, or *PIK3CA* mutations were statistically significant (Fisher’s exact test, RRs: *P* = 0.006, DCRs: *P* = 0.006). On the other hand, there were no statistically significant differences between patients with *KRAS* codon 12 or 13 mutations and those with *KRAS* codon 61, *KRAS* codon 146, *BRAF*, *NRAS*, or *PIK3CA* mutations (Fisher’s exact test, RRs: *P* = 0.636, DCRs: *P* = 0.170).

**Table 4:** Efficacy in the test population determined on the basis of gene status

	**All wild-type (*N*** **= 49)**	***KRAS *codon 12, 13 mutations (*N*** **= 21)**	***KRAS *codon 61, codon 146, *BRAF*, *NRAS *or *PIK3CA *mutations (any other mutations) (*N*** **= 12)**	
Complete response	1	0	0	
Partial response	18	1	0	
Stable disease	19	11	4	
Progressive disease	11	9	8	
Total	49	21	12	
Response rate (%)	38.8	4.8	0	P = 0.006^*^ (All wild-type vs. Any other mutations)
Disease control rate (%)	77.6	57.1	33.3	P = 0.006^*^ (All wild-type vs. Any other mutations)
Progression-free survival [Median (95% CI) (months)]	6.1 (3.1, 9.2)	2.7 (1.2, 4.2)	1.6 (1.5, 1.7)	P < 0.0001^**^ (All wild-type vs. Any other mutations)
Overall survival [Median (95% CI) (months)]	13.8 (9.2, 18.4)	8.2 (5.7, 10.7)	6.3 (1.3, 11.3)	P < 0.0001^**^ (All wild-type vs. Any other mutations)
Relative dose intensity				
Irinotecan [Median (range) (%)]	72.8 (13.0–100)	81.0 (38.4–100)	98.0 (49.3–100)	P = 0.108^***^
Cetuximab [Median (range) (%)]	86.0 (35.7–100)	86.3 (11.1–100)	100 (80.0–100)	P = 0.042^***^
Number of treatment cycles [Median (range)]	12 (1–86)	5 (1–23)	3 (1–12)	P < 0.0001^***^

^*^: Fisher’s exact test.

^**^: log rank test.

^***^: Kruskal–Wallis test.

The relative dose intensity of cetuximab was significantly higher among patients with *KRAS* codon 61, *KRAS* codon 146, *BRAF*, *NRAS*, or *PIK3CA* mutations. However, the number of treatment cycles was significantly greater among patients with all wild-type tumors (Table 4).

RR for all patients included in the study was 24.4%, whereas that for patients with *KRAS* codon 12 or 13 wild-type tumors was 31.1%. Furthermore, RR for patients with all wild-type tumors was 38.8%.

### Survival

**Figure 2:**
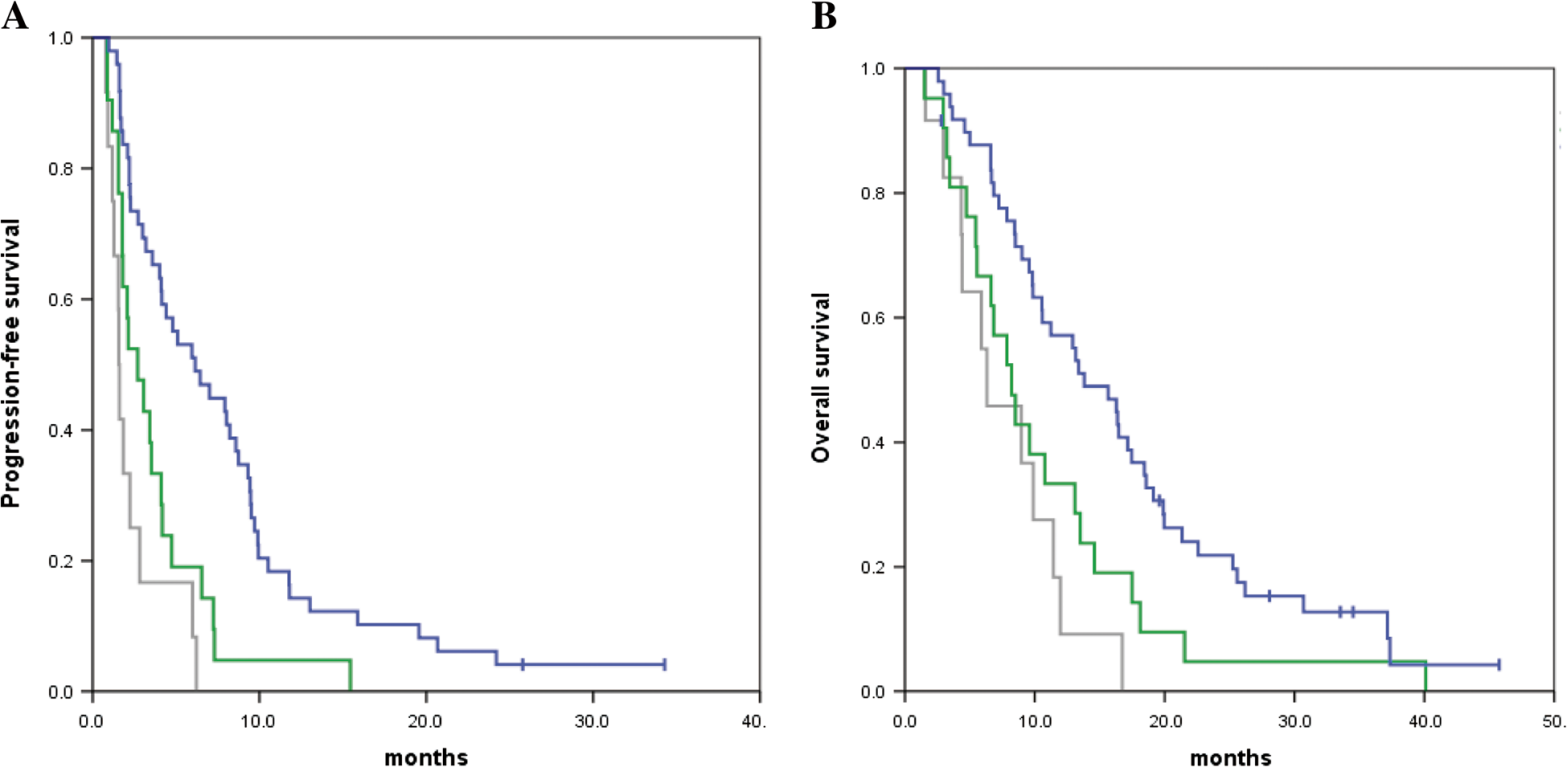
**Kaplan–Meier plots of progression-free survival (PFS) and overall survival (OS) according to *KRAS*, *BRAF*, *NRAS*, and *PIK3CA *gene status. Figure** 2**A. PFS:** Median PFS values were 6.1 months [95% confidence interval (CI): 3.1–9.2], 2.7 months (1.2–4.2), and 1.6 months (1.5–1.7) among patients with all wild-type tumors (*N* = 49, blue line), *KRAS* codon 12 or 13 mutant tumors (*N* = 21, green line), and *KRAS* codon 61, *KRAS* codon 146, *BRAF*, *NRAS*, or *PIK3CA* mutant tumors (*N* = 12, gray-line), respectively. Differences in PFS values between patients with all wild-type tumors and those with *KRAS* codon 61, *KRAS* codon 146, *BRAF*, *NRAS*, or *PIK3CA* mutant tumors were statistically significant (hazard ratio, 0.22; 95% CI, 0.11–0.44; *P* < 0.0001). **Figure** 2**B. OS:** Median OS values were 13.8 months [95% confidence interval (CI): 9.2–18.4], 8.2 months (5.7–10.7), and 6.3 months (1.3–11.3) among patients with all wild-type tumors (*N* = 49, blue line), with *KRAS* codon 12 or 13 mutant tumors (*N* = 21, green line), and with *KRAS* codon 61, *KRAS* codon 146, *BRAF*, *NRAS*, or *PIK3CA* mutations (*N* = 12, gray-line), respectively. Differences in OS values between patients with all wild-type tumors and those with *KRAS* codon 61, *KRAS* codon 146, *BRAF*, *NRAS*, or *PIK3CA* mutant tumors were statistically significant (hazard ratio, 0.30; 95% CI, 0.15–0.61; *P* < 0.0001).

The median PFS among patients with all wild-type tumors (*N* = 49), *KRAS* codon 12 or 13 mutations (*N* = 21), and *KRAS* codon 61, *KRAS* codon 146, *BRAF*, *NRAS*, or *PIK3CA* mutations (*N* = 12) was 6.1 months (95%confidence interval (CI) 3.1–9.2), 2.7 months (1.2–4.2), and 1.6 months (1.5–1.7), respectively (Table 4, Figure 2A). Median OS was 13.8 months (9.2–18.4), 8.2 months (5.7–10.7), and 6.3 months (1.3–11.3), respectively (Table 4, Figure 2B).

We observed statistically significant differences in both PFS and OS between patients with all wild-type tumors and those with *KRAS* codon 61, *KRAS* codon 146, *BRAF*, *NRAS*, or *PIK3CA* mutations [PFS: hazard ratio (HR), 0.22; 95% CI, 0.11–0.44; *P* < 0.0001] (OS: HR, 0.30; 95% CI, 0.15–0.61; *P* < 0.0001) (Figure 2A and 2B). Differences in PFS and OS between patients with wild-type mutations and the 8 patients with *KRAS* codon 61, *KRAS* codon 146, *NRAS*, or *PIK3CA* mutations were statistically significant (PFS: P = 0.001, OS: P = 0.001), but this was not the case for the 4 patients with *BRAF* mutations. The median PFS and OS for these 4 patients were 0.9 months and 11.4 months, respectively.

On the other hand, there were no statistically significant differences between patients with *KRAS* codon 12 or 13 mutations and those with *KRAS* codon 61, *KRAS* codon 146, *BRAF*, *NRAS*, or *PIK3CA* mutations (PFS: *P* = 0.091, OS: *P* = 0.236) (Figure 2A and 2B).

We also analyzed the differences in PFS and OS between patients with *KRAS* codon 12 mutations and those with *KRAS* codon 13 mutations. Similar to our previous study in a different population [17], there were no statistically significant differences between these groups (median PFS: *KRAS* codon 12, 2.1 months vs. *KRAS* codon 13, 3.4 months, P = 0.682; median OS: *KRAS* codon 12, 6.8 months vs. *KRAS* codon 13, 9.6 months, P = 0.147).

## Discussion

This study is the first to verify the relevance of the mutation status of *KRAS* codons 61 and 146, *BRAF*, *NRAS*, and *PIK3CA*to the clinical efficacy of anti-EGFR antibody therapy among Asian patients. As reported in a pooled analysis from a European population, patients with the aforementioned less-frequent mutations exhibited statistically significant worse outcomes equivalent to those of *KRAS* codon 12 and 13 mutants [8]. Though systemically analyzed studies have not been reported since the first European analysis, our results strongly support the usefulness of the expanded pretreatment test for anti-EGFR therapies.

Because our aim was to compare the outcomes of *KRAS* codon 12 and 13 mutant cases with those characterized by other mutations, clinical data and FFPE specimens of the patients treated with cetuximab-containing regimens at seven Japanese cancer centers from July 2008 to April 2010 were collected. At that time, the Japanese authorities did not require pretreatment *KRAS* tests, and patients with *KRAS* codon 12 and 13 mutations were eventually treated with cetuximab. However, the proportion of patients with *KRAS* codon 12 or 13 mutant tumors in this study (25.6%) was slightly lower than that in previous reports of Western and Asian study populations [18], supposedly because several participating institutions had established lab-based tests and used the data for selecting nonbeneficiary populations. Among *KRAS* codon 12 and 13 wild-type cases, the proportion with mutations of overall tested genes (12/61, 19.7%) was similar to that of previous reports, suggesting that such expanded testing would be equally useful in Western and Asian countries.

Because the potential usefulness of multiplex mutation analyses is demonstrated, the development of robust *in vitro* diagnostic systems is needed for clinical application. The application of multiplex mutation detection systems in colorectal cancer specimens has been reported. Lurkin I. et al. reported the validity of multiplex assays using a SNaPshot® Multiplex kit (Life Technologies), which detects 22 mutations in *KRAS*, *BRAF*, *NRAS*, and *PIK3CA*[19]. Here we evaluated a quality-controlled kit detecting 36 mutations of *KRAS* codons 61 and 146, *BRAF*, *NRAS*, and *PIK3CA* using Luminex (xMAP) technology. Data obtained by this kit were fully concordant with those by conventional direct sequencing, regardless of any variation in fixation methods between participating institutes (unpublished data).

This kit has several advantages with regard to its development for routine clinical use. It is manufactured under the same quality as the hitherto approved *in vitro* diagnostic kit detecting mutations in *KRAS* codons 12 and 13. Design of the hands-on operations is simple and easy; detection of the 36 mutations is performed in a single reaction of multiplex PCR followed by Luminex bead assay, with an overall hands-on time of 4.5 h. In addition, the requirement for template DNA is as low as 50 ng. We collected a median of 370 ng (range: 154–889) DNA per 10-μm biopsy slice in this study, which is sufficiently large to perform the test and to reserve backup DNA. Meanwhile, the ARMS–Scorpion assay, another approved *in vitro* diagnostic kit, requires larger amounts of template DNA. The currently approved *KRAS* codons 12 and 13 kit consists of 8 (1 control and 7 mutations) PCR reactions. A total of 80–160 ng of template DNA (10–20 ng for each PCR reaction) are needed to examine a sample [20], and it would be difficult to expand the PCR reactions because of the limitation of template DNA.

It has been estimated that approximately 10%–20% of all patients with colorectal cancer have either *KRAS* codon 61, *KRAS* codon 146, *BRAF*, *NRAS*, or *PIK3CA* gene mutations, suggesting that approximately 60,000–120,000 patients (10%–20% of the 600,000 who die annually from colorectal cancer) worldwide could be screened by this expanded mutation test. Furthermore, because the usefulness of regular administration of aspirin for patients with mutated *PIK3CA* colorectal cancer and the possibility of combining EGFR and BRAF inhibitors for patients with mutated *BRAF* colorectal cancer have been reported, detection of those mutations could become of greater importance in many ways [21,22]. Once further studies with larger sample sizes and a range of clinical samples provide evidence of its clinical utility, this technique might advance the precision of colorectal cancer treatment.

## Conclusions

Our newly developed multiplex kit is practical and feasible for investigating various types of FFPE samples. Moreover, mutations in *KRAS* codon 61, *KRAS* codon 146, *BRAF*, *NRAS*, or *PIK3CA* detected in Asian patients were not predictive of clinical benefits from cetuximab treatment, similar to the result obtained in European studies.

## Abbreviations

EGFR: Anti-epidermal growth factor receptor; PFS: Progression-free survival; OS: Overall survival; CI: Confidence interval; FFPE: Formalin-fixed, paraffin-embedded; CT: Computed tomography; H-E: Hematoxylin–eosin; PCR: Polymerase chain reaction; RR: Response rate; DCR: Disease control rate.

## Competing interests

The authors declare that they have no competing interests.

## Authors’ contributions

TY and KT conceived the study design. HB carried out the majority of molecular genetic studies and analyses of the clinical data. ES, TN, KY, KY, SY, and SK provided clinical data and helped collect tumor tissues. SF carried out the pathological diagnoses. TY statistically analyzed the clinical data. AO coordinated the study and helped to draft the manuscript. All authors have read and approved the final manuscript.

## Pre-publication history

The pre-publication history for this paper can be accessed here:

http://www.biomedcentral.com/1471-2407/13/405/prepub

## Research group members

Hideaki Bando, Takayuki Yoshino, Katsuya Tsuchihara, Satoshi Fujii, Kohei Shitara, Takeharu Yamanaka, and Atsushi Ohtsu (National Cancer Center Hospital East); Satoshi Yuki and Takahide Sasaki (Hokkaido University); Eiji Shinozaki (Cancer Institute Hospital of Japanese Foundation for Cancer Research); Tomohiro Nishina (Shikoku Cancer Center); Kensei Yamaguchi, Shigenori Kadowaki, and Masako Asayama (Saitama Cancer Center); Kentaro Yamazaki (Shizuoka Cancer Center) and Shinya Kajiura (University of Toyama).
